# Crystal structure and Hirshfeld surface analysis of 2,6-bis­[1-(prop-2-yn-1-yl)-1*H*-benzo[*d*]imidazol-2-yl]pyridine 0.144-hydrate

**DOI:** 10.1107/S2056989025009740

**Published:** 2025-11-11

**Authors:** Mohamed Ait Idar, Yousri El Barkaoui, Tuncer Hökelek, Olivier Blacque, Hassan Cherkaoui, Ahmed Moussaif

**Affiliations:** a Laboratory of Heterocyclic Organic Chemistry Medicines Science Research Center Pharmacochemistry Competence Center Mohammed V University in Rabat Faculté des Sciences Av Ibn Battouta BP 1014 Rabat Morocco; b Regional Center for Education and Training Professions in the Rabat Region, Morocco; c Department of Physics, Hacettepe University, 06800 Beytepe, Ankara, Türkiye; d University of Zurich, Department of Chemistry B, Winterthurerstrasse 190, 8057 Zurich, Switzerland; e National Center for Nuclear Energy, Science and Technology,Rabat Morocco; Vienna University of Technology, Austria

**Keywords:** Benzo[*d*]imidazol, crystal structure, π-stacking, hydrogen bond

## Abstract

The title compound, C_25_H_17_N_5_·0.144H_2_O, contains two substituted benzimidazole ring systems bridged over a pyridine ring and a disordered non-coord­inating water mol­ecule. In the crystal, O—H⋯N and C—H⋯O hydrogen-bonding inter­actions link the mol­ecules into infinite chains parallel to [101]. Furthermore, π–π stacking inter­actions between the imidazole rings and between the pyridine and imidazole rings of adjacent mol­ecules with inter-centroid distances of 3.6371 (4), 3.9872 (5), 3.4916 (4) and 3.6648 (4) Å are present, but C—H⋯π(ring) inter­actions are not observed. Hirshfeld surface analysis of the crystal structure indicates that the most important contributions for the crystal packing are from H⋯H (39.3%), H⋯C/C⋯H (35.9%) and H⋯N/N⋯H (9.1%).

## Abstract 2

In the hydrated title compound, two substituted benzimidazole ring systems are bridged over a pyridine ring. O—H—N and C—H—O hydrogen bonds as well as π–π stacking inter­actions consolidate the crystal packing.

## 1. Chemical context

Heterocyclic compounds occupy a central position in medicinal chemistry as they are key elements in the search for the and development of new bioactive mol­ecules for the pharmaceutical industry (Vitaku *et al.*, 2014 ▸). Among them, nitro­gen-containing heterocycles exhibit a wide range of biological activities due to their structural similarities with numerous natural and synthetic mol­ecules already known for their pharmacological properties (Tahlan *et al.*, 2019 ▸). In this context, benzimidazole is a noteworthy representative and an important pharmacophore and scaffold in medicinal chemistry (Al-Ghulikah *et al.*, 2023 ▸). This core structure is frequently employed as a basis for designing therapeutic mol­ecules of pharmaceutical and biological inter­ests. Benzimidazole derivatives have demonstrated a broad spectrum of biological activities, including anti­histaminic, anti­ulcer, anti­bacterial, anti­parasitic, anti­cancer, anti­viral, anti-inflammatory, anti­oxidant and anti­diabetic properties (Saber *et al.*, 2021 ▸; Leonard *et al.*, 2006 ▸; Reddy *et al.*, 2005 ▸).

Building on our previous work on benzimidazole-based systems (Missioui *et al.*, 2022 ▸; Moussaif *et al.*, 2025 ▸), we now report the synthesis, structure and Hirshfeld surface of the title compound, C_25_H_17_N_5_·0.144H_2_O. The mol­ecular and crystal structure of this compound was established unambiguously by single-crystal X-ray diffraction. To gain deeper insight into its supra­molecular features, a Hirshfeld surface analysis was undertaken, which enabled the identification and qu­anti­fication of the key inter­molecular inter­actions governing the organization of the crystal structure.
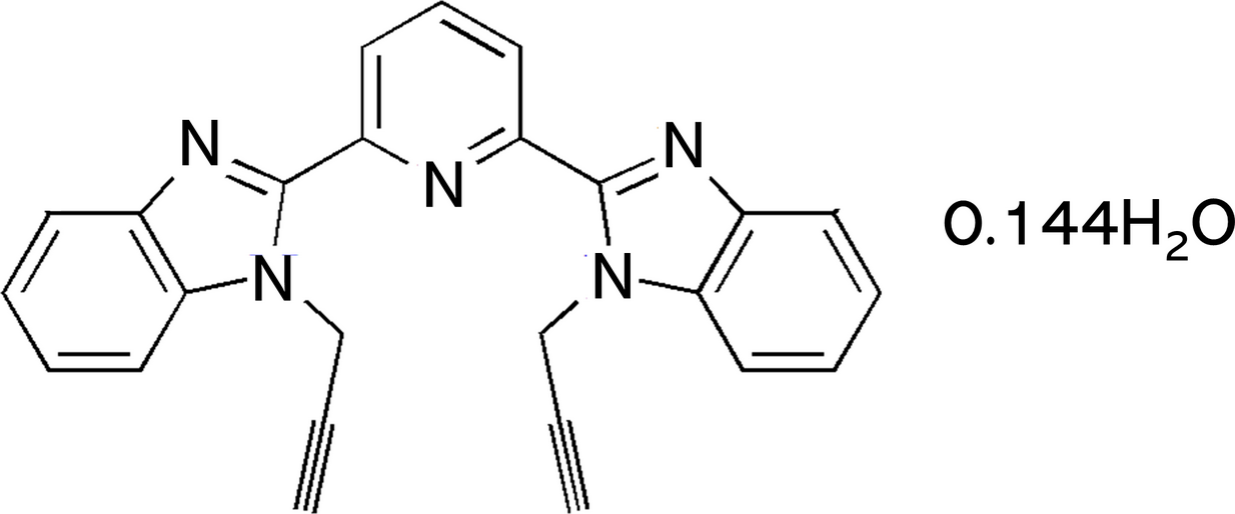


## 2. Structural commentary

**Figure 1:**
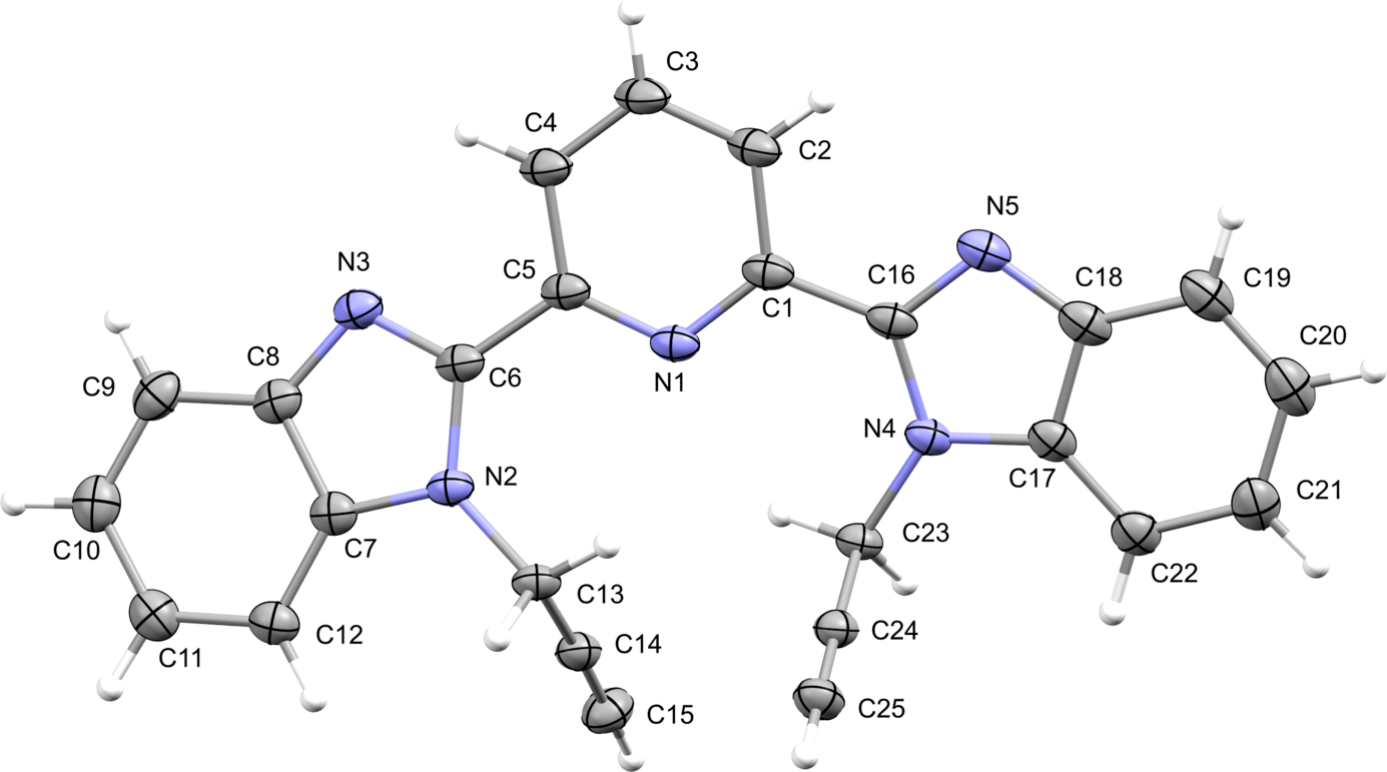
The mol­ecular structure of the title compound with displacement ellipsoids drawn at the 50% probability level. The disordered water mol­ecule is not shown for clarity.

The title compound contains two benzimidazole entities bridged over a pyridine ring, two propyl moieties and a disordered non-coordinating water mol­ecule (Fig. 1 ▸). The dihedral angles between the imidazole rings (*B*, N2/N3/C6–C8; *D*, N4/N5/C16–C18) and the benzene rings (*C*, C7–C12; *E*, C17–C22) of the heterocyclic moieties, are *B*/*C* = 1.93 (4)° and *D*/*E* = 0.97 (5)°. Thus, the two benzimidazole rings are almost planar. The central pyridine ring (*A*, N1/C1–C5) is oriented at dihedral angles of 11.77 (4) and 6.64 (3)°, respectively, to the mean plane of the benzimidazole rings *B* and *D*. The dihedral angle between the two benzimidazole ring systems *BD* and *DE* is 18.26 (3)°. There are no unusual bond lengths or inter­bond angles in the mol­ecule.

## 3. Supra­molecular features

**Figure 2:**
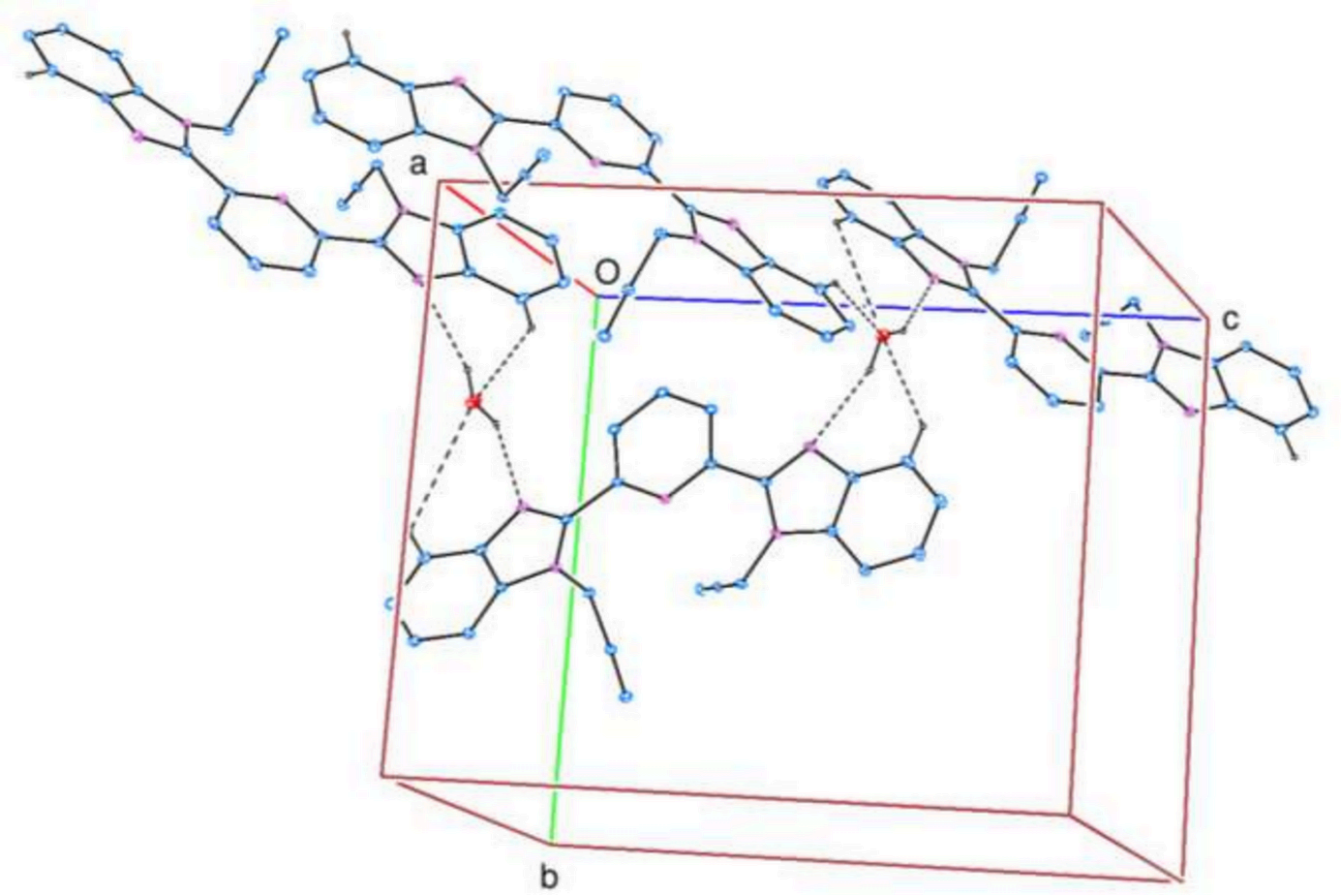
A partial packing diagram of the title compound with inter­molecular O—H⋯N and C—H⋯O hydrogen bonds shown as dashed lines. Hydrogen atoms not involved in these inter­actions have been omitted.

In the crystal, O—H⋯N and C—H⋯O hydrogen-bonding inter­actions (Table 1 ▸) between the non-coordinating water mol­ecule and the benzimidazole rings link mol­ecules into infinite chains extending parallel to [101] (Fig. 2 ▸). Furthermore, π–π stacking inter­actions between the *B* rings [centroid-to-centroid distance = 3.6371 (4) Å, α = 0.04 (3)°, slippage = 1.150 Å], *B* and *D* rings [centroid-to-centroid distance = 3.9872 (5) Å, α = 1.34 (5)°, slippage = 1.957 Å], *D* rings [centroid-to-centroid distance = 3.4916 (4) Å, α = 0.00 (5)°, slippage = 0.947 Å] and *A* and *E* rings [centroid-to-centroid distance = 3.6648 (4) Å, α = 6.81 (5)°, slippage = 1.461 Å] of adjacent mol­ecules may help to consolidate the packing. C—H⋯π(ring) inter­actions are not observed.

## 4. Hirshfeld surface analysis

**Figure 3:**
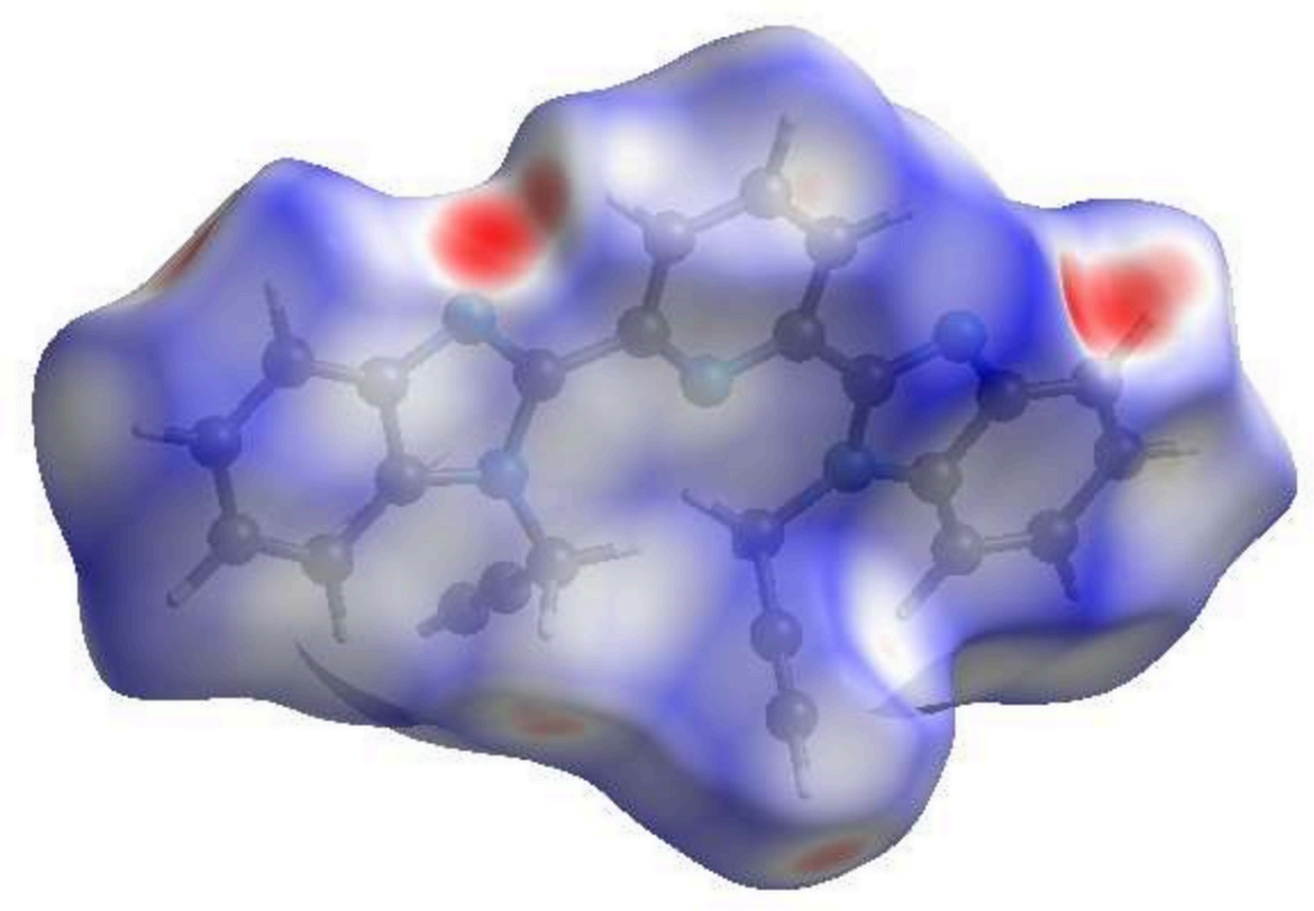
View of the three-dimensional Hirshfeld surface plotted over *d*_norm_.

**Figure 4:**
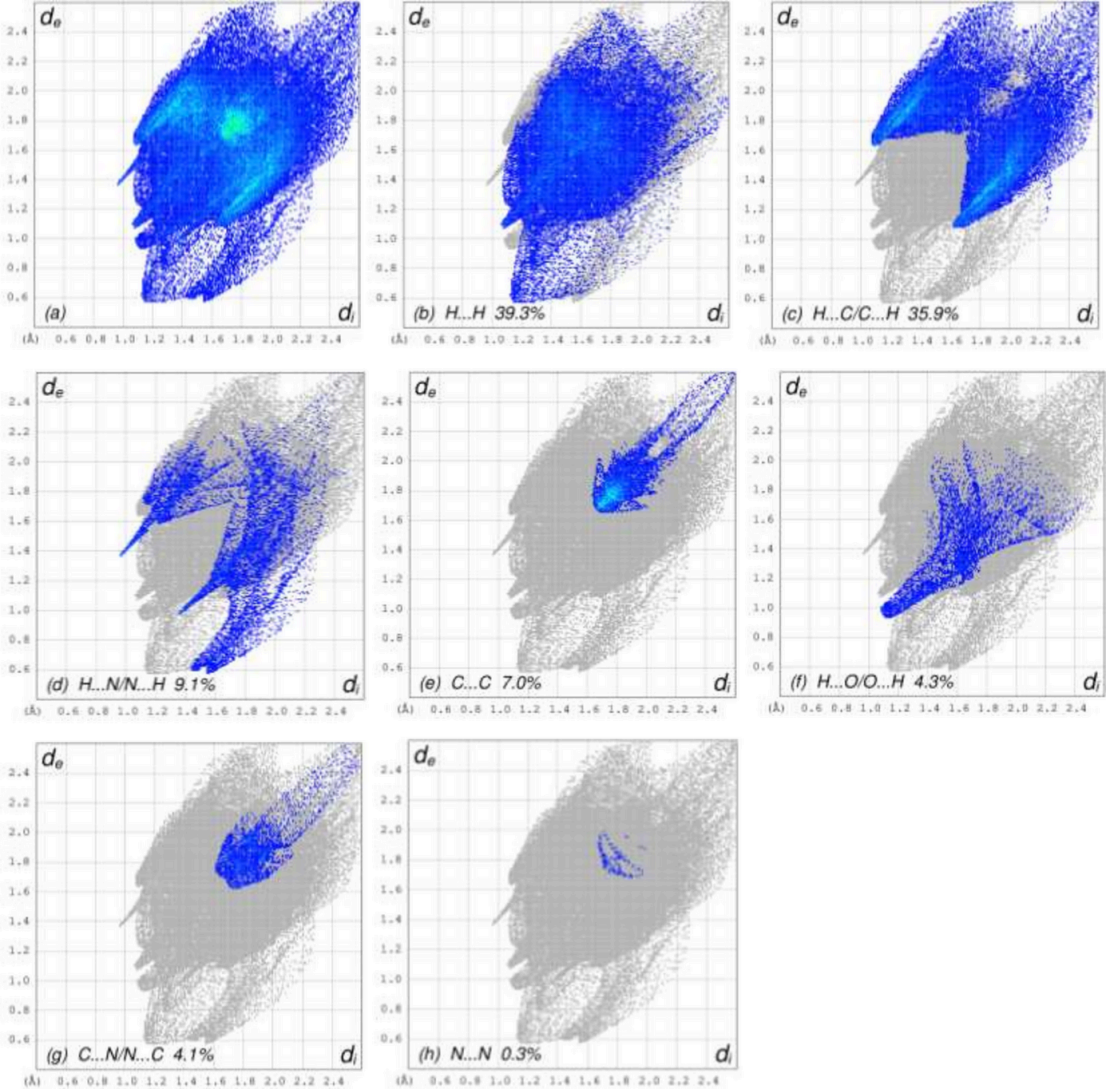
Two-dimensional fingerprint plots, showing (*a*) all inter­actions, and delineated into (*b*) H⋯H, (*c*) H⋯C/C⋯H, (*d*) H⋯N/N⋯H, (*e*) C⋯C, (*f*) H⋯O/O⋯H, (*g*) C⋯N/N⋯C and (*h*) N⋯N inter­actions. The *d*_i_ and *d*_e_ values are the closest inter­nal and external distances (in Å) from given points on the Hirshfeld surface contacts.

In order to visualize and qu­antify the inter­molecular inter­actions in the crystal, a Hirshfeld surface (HS) analysis was carried out using *CrystalExplorer* (Spackman *et al.*, 2021 ▸) following the protocol of Tan *et al.* (2019 ▸) after non-consideration of the partially occupied water molecule. Fig. 3 ▸ shows the contact distances where the bright-red spots correspond to the respective donors and/or acceptors noted above; numerical values of contact distances are collated in Table 2 ▸. According to the two-dimensional fingerprint plots (McKinnon *et al.*, 2007 ▸), the H⋯H, H⋯ C/C⋯H and H⋯N/N⋯H contacts make the most significant contributions to the HS, at 39.3%, 35.9%, 14.5% and 9.1%, respectively (Table 2 ▸, Fig. 4 ▸).

## 5. Database survey

**Figure 5:**
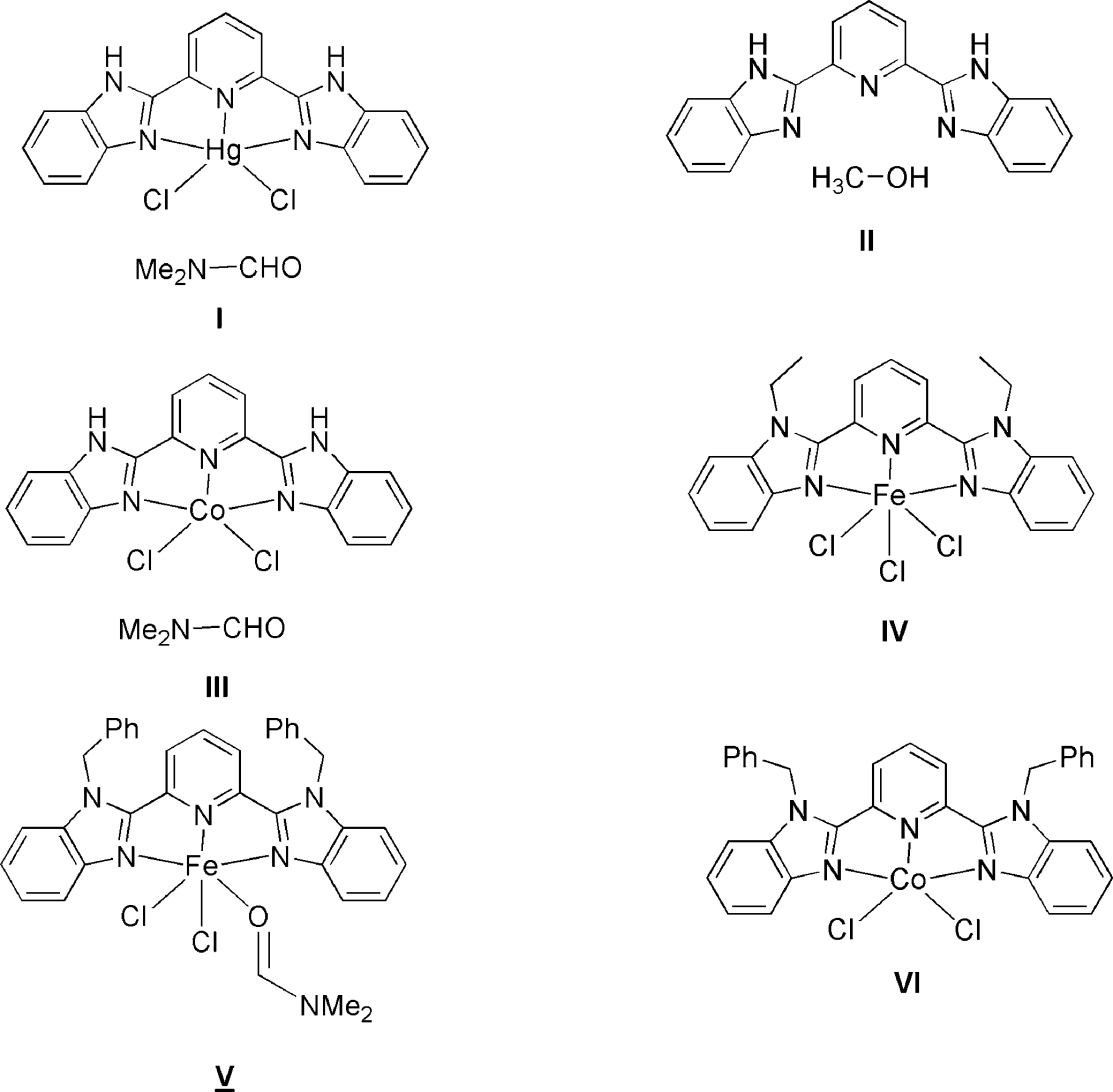
Structures closely related to the title compound according to a CSD search.

A search of the Cambridge Structural Database (CSD, July 2025 update; Groom *et al.*, 2016 ▸) revealed several entries closely related to the title compound, a derivative of 2-(6-(1*H*-benzo[*d*]imidazol-2-yl)pyridin-2-yl)-1*H*-benzo[*d*]imidazole. The most relevant analogs are illustrated in Fig. 5 ▸ and include compounds **I** (CSD refcode DIXNUU; Liu *et al.*, 2007 ▸), **II** (MOTGEI; Chen *et al.*, 2009 ▸), **III** (WAKJID01; Gong *et al.*, 2012 ▸), **IV** (VAPTEN; Gong *et al.*, 2012 ▸), **V** (VAPTEN; Gong *et al.*, 2012 ▸), and **VI** (VAPVEP; Gong *et al.*, 2012 ▸). A detailed comparative analysis of these structures and the title compound highlights both common structural characteristics and distinctive features.

*Core mol­ecular geometry*. All compounds display a benzimidazole–pyridine–benzimidazole framework that remains essentially planar. Compounds **I**–**III**, exhibit classical N—H⋯N or N—H⋯O hydrogen bonds between the constituents, which leads to the formation of supra­molecular chains or layers. In the title compound, however, the hydrogen-bonding network is more compact and directional, leading to a more compact packing arrangement than in the other analogues.

*Influence of metal coordination*. Compounds **IV**–**VI** feature coordination to metals, which significantly alters both structural details within the organic ligands and in the supra­molecular packing modes.

*Crystal packing and π–π stacking*. In all of the above related structures, π–π stacking appears to play a significant role in consolidating the crystal structure. Notably, the title compound exhibits slightly shorter centroid-to-centroid distances compared to its metal-coordinating analogues, indicating stronger inter­molecular π–π inter­actions. This comparative structural analysis demonstrates that the title compound, although it shares a common mol­ecular framework with its analogues, has a unique supra­molecular organization that is governed by the absence of metal coordination and the predominance of hydrogen-bonding and π–π stacking inter­actions. These observations provide insight into the impact of structural modifications on mol­ecular packing and overall crystal architectures.

## 6. Synthesis and crystallization

**Figure 6:**
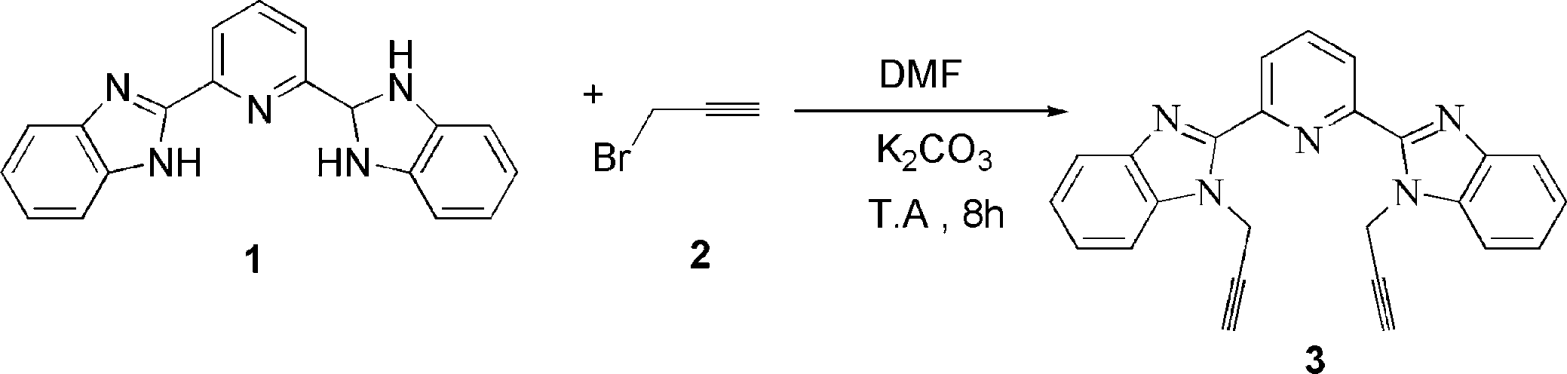
Reaction scheme for obtaining the title compound.

The synthesis of the title compound is shown schematically in Fig. 6 ▸. In a 100 ml round-bottom flask, 2,6-bis­(1*H*-benzo[*d*]imidazol-2-yl)pyridine (**1**) (0.30 g, 0.96 mmol) was combined with potassium carbonate (K_2_CO_3_) (0.34 g, 2.49 mmol) in 10 ml of dimethylformamide (DMF). The mixture was stirred at room temperature for 15 min to ensure homogenization. Subsequently, propargyl bromide (**2**) (0.20 ml, 2.30 mmol) was added dropwise under continuous stirring. The progress of the reaction was monitored by thin-layer chromatography (TLC), and stirring was continued for 8 h at room temperature. After completion, the solvent was removed under reduced pressure using a rotary evaporator. The resulting crude residue was extracted with ethyl acetate and water. The organic layer was collected, dried over anhydrous sodium sulfate, and filtered. Purification by recrystallization from ethanol solution afforded the title compound (**3**) in 75% yield (Fig. 6 ▸). ^1^H NMR (500 MHz, DMSO-*d*_6_) δ (ppm): 9.30 (*d*; *J* = 7.9 Hz; 2H; CH_pyr_); 9.11 (*dd*; *J* = 8.2, 7.6 Hz; 1H; CH_pyr_); 8.69–8.20 (*m*; 8H; CH_Ar_); 6.68 (*d*; 4H; *J* = 2.5 Hz; –NCH_2_); 4.23 (*t*; 2H; –CCH).

## 7. Refinement

Crystal data, data collection and structure refinement details are summarized in Table 3 ▸. After refinement of the organic mol­ecule (*R*1/*wR*2 = 0.0528/0.1440), relatively high residual electron density was located between mol­ecules, pointing to an underoccupied oxygen atom of a water mol­ecule. Additional positional disorder using SIMU restraints was introduced, and the occupancies independently refined. The ‘add-H′ tool in *OLEX2* (Dolomanov *et al.*, 2009 ▸) was used for the H atoms of the water mol­ecules, with a fixed O—H distance of 0.87 Å and *U*_iso_(H) = 1.5*U*_eq_(O). Other hydrogen atom positions were calculated geometrically at CH = 0.95 Å and CH_2_ = 0.99 Å and refined using a riding model with *U*_iso_(H) = 1.2*U*_eq_(C).

## Supplementary Material

Crystal structure: contains datablock(s) I. DOI: 10.1107/S2056989025009740/wm5772sup1.cif

Structure factors: contains datablock(s) I. DOI: 10.1107/S2056989025009740/wm5772Isup2.hkl

Supporting information file. DOI: 10.1107/S2056989025009740/wm5772Isup3.cdx

Supporting information file. DOI: 10.1107/S2056989025009740/wm5772Isup4.cdx

Supporting information file. DOI: 10.1107/S2056989025009740/wm5772Isup5.cml

CCDC reference: 2500024

Additional supporting information:  crystallographic information; 3D view; checkCIF report

## Figures and Tables

**Table 1:** Hydrogen-bond geometry (Å, °)

*D*—H⋯*A*	*D*—H	H⋯*A*	*D*⋯*A*	*D*—H⋯*A*
O1*A*—H1*A*⋯N3^iv^	0.87	2.14	2.923 (16)	149
O1*A*—H1*B*⋯N5	0.87	2.37	3.178 (17)	154
O1*B*—H1*C*⋯N3^iv^	0.87	2.21	2.96 (3)	144
O1*B*—H1*D*⋯N5	0.87	2.24	3.07 (4)	161
C9—H9⋯O1*A*^i^	0.95	2.16	2.891 (15)	133
C9—H9⋯O1*B*^i^	0.95	2.15	2.93 (3)	139
C19—H19⋯O1*A*	0.95	2.36	3.086 (18)	133

Symmetry codes: (i) 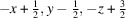; (iv) 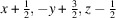.

**Table 2:** Selected interatomic distances (Å)

H9⋯O1*A*^i^	2.16	C12⋯C14	3.376 (2)
H4⋯O1*A*^ii^	2.62	C16⋯C17^vi^	3.3783 (19)
O1*A*⋯H19	2.36	C1⋯H23*B*	2.89
O1*B*⋯H1*D*	0.87	H1*A*⋯C4^iv^	2.85
H9⋯O1*B*^i^	2.15	C5⋯H13*B*	2.87
H13*A*⋯O1*B*^iii^	2.37	C13⋯H23*B*	2.86
O1*B*⋯H1*C*	0.87	C14⋯H23*B*	2.78
N1⋯N4	2.9842 (16)	C18⋯H1*B*	2.56
N1⋯C13	3.0587 (18)	C18⋯H1*D*	2.60
N1⋯N2	3.0092 (16)	C19⋯H1*B*	2.38
N1⋯C23	3.012 (2)	C19⋯H1*D*	2.64
N1⋯H23*B*	2.34	C22⋯H23*A*	2.87
N1⋯H13*B*	2.36	C23⋯H13*B*	2.85
H1*A*⋯N3^iv^	2.14	C24⋯H13*B*	2.74
N3⋯H25^v^	2.46	H1*A*⋯H19	2.37
H1*C*⋯N3^iv^	2.21	H4⋯H1*A*^ii^	1.96
N3⋯H4	2.44	H1*B*⋯H19	1.83
N5⋯H2	2.45	H4⋯H1*C*^ii^	2.37
N5⋯H1*B*	2.37	H1*C*⋯H1*D*	1.38
N5⋯H1*D*	2.24	H1*D*⋯H19	2.21
C2⋯C22^vi^	3.381 (2)	H13*B*⋯H23*B*	2.19
C5⋯C21^vi^	3.392 (2)	H19⋯H1*A*	2.37
C5⋯C20^vi^	3.386 (2)		

Symmetry codes: (i) 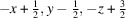; (ii) 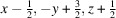; (iii) 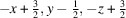; (iv) 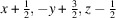; (v) 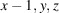; (vi) 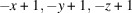.

**Table 3:** Experimental details

Crystal data	
Chemical formula	C_25_H_17_N_5_·0.144H_2_O
*M*_r_	390.04
Crystal system, space group	Monoclinic, *P*2_1_/*n*
Temperature (K)	160
*a*, *b*, *c* (Å)	9.6858 (2), 13.7005 (2), 15.0908 (3)
β (°)	102.943 (2)
*V* (Å^3^)	1951.68 (6)
*Z*	4
Radiation type	Cu *K*α
μ (mm^−1^)	0.65
Crystal size (mm)	0.22 × 0.15 × 0.07

Data collection	
Diffractometer	XtaLAB Synergy, Dualflex, HyPix
Absorption correction	Analytical [*CrysAlis PRO* (Rigaku OD, 2024 ▸) using a multifaceted crystal model (Clark & Reid, 1995 ▸)]
*T*_min_, *T*_max_	0.900, 0.965
No. of measured, independent and observed [*I* > 2σ(*I*)] reflections	21779, 4122, 3852
*R*_int_	0.022
(sin θ/λ)_max_ (Å^−1^)	0.633

Refinement	
*R*[*F*^2^ > 2σ(*F*^2^)], *wR*(*F*^2^), *S*	0.043, 0.102, 1.07
No. of reflections	4122
No. of parameters	298
No. of restraints	6
H-atom treatment	H-atom parameters constrained
Δρ_max_, Δρ_min_ (e Å^−3^)	0.26, −0.17

Computer programs: *CrysAlis PRO* (Rigaku OD, 2024 ▸), *SHELXT* (Sheldrick, 2015*a* ▸), *SHELXL* (Sheldrick, 2015*b* ▸), *OLEX2* (Dolomanov *et al.*, 2009 ▸) and *publCIF* (Westrip, 2010 ▸).

## supplementary crystallographic information

### 2,6-Bis[1-(prop-2-yn-1-yl)-1H-benzo[d]imidazol-2-yl]pyridine 0.144-hydrate Crystal data

**Table:**

C_25_H_17_N_5_·0.144H_2_O	*F*(000) = 814
*M**_r_* = 390.04	*D*_x_ = 1.327 Mg m^−^^3^
Monoclinic, *P*2_1_/*n*	Cu *K*α radiation, λ = 1.54184 Å
*a* = 9.6858 (2) Å	Cell parameters from 13938 reflections
*b* = 13.7005 (2) Å	θ = 3.0–78.9°
*c* = 15.0908 (3) Å	µ = 0.65 mm^−^^1^
β = 102.943 (2)°	*T* = 160 K
*V* = 1951.68 (6) Å^3^	Plate, colourless
*Z* = 4	0.22 × 0.15 × 0.07 mm

### 2,6-Bis[1-(prop-2-yn-1-yl)-1H-benzo[d]imidazol-2-yl]pyridine 0.144-hydrate Data collection

**Table:**

XtaLAB Synergy, Dualflex, HyPix diffractometer	4122 independent reflections
Radiation source: micro-focus sealed X-ray tube, PhotonJet (Cu) X-ray Source	3852 reflections with *I* > 2σ(*I*)
Mirror monochromator	*R*_int_ = 0.022
Detector resolution: 10.0000 pixels mm^-1^	θ_max_ = 77.4°, θ_min_ = 4.4°
ω scans	*h* = −12→12
Absorption correction: analytical [CrysAlisPro (Rigaku OD, 2024) using a multifaceted crystal model (Clark & Reid, 1995)]	*k* = −16→17
*T*_min_ = 0.900, *T*_max_ = 0.965	*l* = −19→17
21779 measured reflections	

### 2,6-Bis[1-(prop-2-yn-1-yl)-1H-benzo[d]imidazol-2-yl]pyridine 0.144-hydrate Refinement

**Table:**

Refinement on *F*^2^	Hydrogen site location: mixed
Least-squares matrix: full	H-atom parameters constrained
*R*[*F*^2^ > 2σ(*F*^2^)] = 0.043	*w* = 1/[σ^2^(*F*_o_^2^) + (0.0359*P*)^2^ + 0.7695*P*] where *P* = (*F*_o_^2^ + 2*F*_c_^2^)/3
*wR*(*F*^2^) = 0.102	(Δ/σ)_max_ = 0.001
*S* = 1.07	Δρ_max_ = 0.26 e Å^−^^3^
4122 reflections	Δρ_min_ = −0.16 e Å^−^^3^
298 parameters	Extinction correction: SHELXL-2019/2 (Sheldrick, 2015b), Fc^*^=kFc[1+0.001xFc^2^λ^3^/sin(2θ)]^-1/4^
6 restraints	Extinction coefficient: 0.00117 (14)
Primary atom site location: dual	

### 2,6-Bis[1-(prop-2-yn-1-yl)-1H-benzo[d]imidazol-2-yl]pyridine 0.144-hydrate Special details

**Table:**

Geometry. All esds (except the esd in the dihedral angle between two l.s. planes) are estimated using the full covariance matrix. The cell esds are taken into account individually in the estimation of esds in distances, angles and torsion angles; correlations between esds in cell parameters are only used when they are defined by crystal symmetry. An approximate (isotropic) treatment of cell esds is used for estimating esds involving l.s. planes.

### 2,6-Bis[1-(prop-2-yn-1-yl)-1H-benzo[d]imidazol-2-yl]pyridine 0.144-hydrate Fractional atomic coordinates and isotropic or equivalent isotropic displacement parameters (Å^2^)

**Table:**

	*x*	*y*	*z*	*U*_iso_*/*U*_eq_	Occ. (<1)
O1A	0.5569 (16)	0.8667 (13)	0.4309 (17)	0.053 (5)	0.094 (6)
H1A	0.631531	0.888804	0.414429	0.079*	0.094 (6)
H1B	0.580600	0.808201	0.451140	0.079*	0.094 (6)
O1B	0.586 (3)	0.877 (3)	0.479 (3)	0.055 (6)	0.050 (5)
H1C	0.649154	0.890204	0.448456	0.083*	0.050 (5)
H1D	0.586936	0.813222	0.483298	0.083*	0.050 (5)
N1	0.46815 (11)	0.54778 (8)	0.72961 (8)	0.0360 (3)	
N2	0.47168 (11)	0.42143 (8)	0.89321 (8)	0.0353 (2)	
N3	0.26310 (12)	0.49018 (9)	0.89744 (8)	0.0422 (3)	
N4	0.64889 (11)	0.52420 (8)	0.59477 (8)	0.0356 (2)	
N5	0.55002 (13)	0.66248 (9)	0.52978 (8)	0.0437 (3)	
C1	0.46243 (14)	0.61188 (10)	0.66146 (10)	0.0386 (3)	
C2	0.36641 (17)	0.68994 (11)	0.64552 (11)	0.0476 (4)	
H2	0.367614	0.735284	0.598073	0.057*	
C3	0.27084 (18)	0.69972 (12)	0.69953 (11)	0.0518 (4)	
H3	0.204134	0.751643	0.689551	0.062*	
C4	0.27245 (17)	0.63337 (11)	0.76859 (10)	0.0477 (4)	
H4	0.206054	0.638148	0.806157	0.057*	
C5	0.37380 (14)	0.55902 (10)	0.78210 (9)	0.0382 (3)	
C6	0.37072 (14)	0.49008 (10)	0.85648 (9)	0.0372 (3)	
C7	0.42394 (14)	0.37547 (10)	0.96244 (9)	0.0372 (3)	
C8	0.29418 (15)	0.41936 (10)	0.96437 (10)	0.0404 (3)	
C9	0.21911 (17)	0.39074 (12)	1.02873 (11)	0.0485 (4)	
H9	0.130887	0.419885	1.030806	0.058*	
C10	0.27767 (18)	0.31844 (12)	1.08943 (11)	0.0509 (4)	
H10	0.229306	0.298193	1.134536	0.061*	
C11	0.40679 (17)	0.27434 (11)	1.08590 (11)	0.0480 (4)	
H11	0.443327	0.224444	1.128418	0.058*	
C12	0.48269 (15)	0.30134 (11)	1.02228 (10)	0.0427 (3)	
H12	0.569882	0.271054	1.019571	0.051*	
C13	0.60936 (13)	0.39938 (10)	0.87291 (10)	0.0368 (3)	
H13A	0.683742	0.402283	0.929774	0.044*	
H13B	0.631548	0.449353	0.830796	0.044*	
C14	0.61060 (14)	0.30259 (10)	0.83176 (10)	0.0391 (3)	
C15	0.61316 (17)	0.22284 (12)	0.80235 (12)	0.0507 (4)	
H15	0.615212	0.158724	0.778700	0.061*	
C16	0.55498 (14)	0.59963 (10)	0.59699 (9)	0.0378 (3)	
C17	0.70860 (14)	0.54168 (10)	0.52136 (9)	0.0387 (3)	
C18	0.64509 (15)	0.62793 (11)	0.48159 (10)	0.0429 (3)	
C19	0.68005 (17)	0.66414 (12)	0.40296 (11)	0.0511 (4)	
H19	0.636823	0.721620	0.374186	0.061*	
C20	0.77899 (17)	0.61388 (13)	0.36863 (11)	0.0547 (4)	
H20	0.804802	0.637632	0.315445	0.066*	
C21	0.84308 (16)	0.52855 (13)	0.40971 (11)	0.0503 (4)	
H21	0.911830	0.496311	0.384139	0.060*	
C22	0.80837 (15)	0.49030 (12)	0.48667 (10)	0.0445 (3)	
H22	0.850488	0.431997	0.514347	0.053*	
C23	0.68304 (13)	0.43763 (9)	0.65209 (9)	0.0353 (3)	
H23A	0.688719	0.380165	0.613293	0.042*	
H23B	0.606340	0.425733	0.684480	0.042*	
C24	0.81836 (14)	0.44900 (10)	0.71894 (9)	0.0377 (3)	
C25	0.92809 (15)	0.45846 (12)	0.77189 (11)	0.0470 (4)	
H25	1.016051	0.466048	0.814336	0.056*	

### 2,6-Bis[1-(prop-2-yn-1-yl)-1H-benzo[d]imidazol-2-yl]pyridine 0.144-hydrate Atomic displacement parameters (Å^2^)

**Table:**

	*U*^11^	*U*^22^	*U*^33^	*U*^12^	*U*^13^	*U*^23^
O1A	0.027 (7)	0.053 (7)	0.080 (13)	0.000 (5)	0.016 (8)	0.010 (9)
O1B	0.032 (10)	0.053 (10)	0.081 (15)	0.001 (8)	0.014 (11)	0.002 (13)
N1	0.0326 (5)	0.0309 (5)	0.0393 (6)	0.0027 (4)	−0.0030 (4)	−0.0031 (5)
N2	0.0305 (5)	0.0328 (5)	0.0391 (6)	0.0029 (4)	0.0006 (4)	−0.0035 (5)
N3	0.0374 (6)	0.0421 (6)	0.0450 (6)	0.0055 (5)	0.0046 (5)	−0.0088 (5)
N4	0.0299 (5)	0.0328 (5)	0.0404 (6)	−0.0006 (4)	0.0000 (4)	0.0046 (5)
N5	0.0413 (6)	0.0350 (6)	0.0482 (7)	−0.0020 (5)	−0.0036 (5)	0.0063 (5)
C1	0.0368 (7)	0.0310 (6)	0.0416 (7)	0.0028 (5)	−0.0047 (5)	−0.0025 (5)
C2	0.0534 (9)	0.0372 (7)	0.0456 (8)	0.0131 (6)	−0.0030 (7)	0.0011 (6)
C3	0.0571 (9)	0.0442 (8)	0.0480 (8)	0.0242 (7)	−0.0012 (7)	−0.0042 (7)
C4	0.0480 (8)	0.0453 (8)	0.0452 (8)	0.0172 (7)	0.0005 (6)	−0.0066 (6)
C5	0.0357 (7)	0.0348 (7)	0.0395 (7)	0.0062 (5)	−0.0014 (5)	−0.0075 (5)
C6	0.0331 (6)	0.0345 (6)	0.0395 (7)	0.0050 (5)	−0.0014 (5)	−0.0091 (5)
C7	0.0360 (7)	0.0343 (7)	0.0382 (7)	−0.0034 (5)	0.0015 (5)	−0.0075 (5)
C8	0.0385 (7)	0.0374 (7)	0.0423 (7)	−0.0009 (6)	0.0032 (6)	−0.0108 (6)
C9	0.0456 (8)	0.0477 (8)	0.0539 (9)	−0.0032 (6)	0.0145 (7)	−0.0141 (7)
C10	0.0580 (9)	0.0463 (8)	0.0501 (9)	−0.0118 (7)	0.0159 (7)	−0.0094 (7)
C11	0.0546 (9)	0.0417 (8)	0.0453 (8)	−0.0078 (7)	0.0058 (7)	−0.0020 (6)
C12	0.0410 (7)	0.0382 (7)	0.0449 (8)	−0.0012 (6)	0.0014 (6)	−0.0020 (6)
C13	0.0286 (6)	0.0339 (6)	0.0443 (7)	0.0026 (5)	0.0010 (5)	0.0000 (6)
C14	0.0332 (6)	0.0384 (7)	0.0448 (7)	0.0050 (5)	0.0069 (6)	0.0031 (6)
C15	0.0524 (9)	0.0385 (8)	0.0637 (10)	0.0051 (7)	0.0185 (8)	−0.0041 (7)
C16	0.0332 (6)	0.0307 (6)	0.0430 (7)	−0.0011 (5)	−0.0051 (5)	0.0017 (5)
C17	0.0305 (6)	0.0407 (7)	0.0399 (7)	−0.0081 (5)	−0.0024 (5)	0.0038 (6)
C18	0.0372 (7)	0.0387 (7)	0.0455 (8)	−0.0104 (6)	−0.0060 (6)	0.0067 (6)
C19	0.0462 (8)	0.0495 (9)	0.0505 (9)	−0.0148 (7)	−0.0041 (7)	0.0135 (7)
C20	0.0472 (9)	0.0660 (10)	0.0455 (8)	−0.0238 (8)	−0.0008 (7)	0.0105 (8)
C21	0.0363 (7)	0.0656 (10)	0.0463 (8)	−0.0139 (7)	0.0036 (6)	0.0009 (7)
C22	0.0335 (7)	0.0503 (8)	0.0459 (8)	−0.0062 (6)	0.0009 (6)	0.0041 (7)
C23	0.0325 (6)	0.0296 (6)	0.0410 (7)	0.0018 (5)	0.0022 (5)	0.0040 (5)
C24	0.0340 (7)	0.0351 (7)	0.0430 (7)	0.0042 (5)	0.0064 (6)	0.0048 (6)
C25	0.0358 (7)	0.0521 (9)	0.0488 (8)	0.0023 (6)	0.0006 (6)	0.0066 (7)

### 2,6-Bis[1-(prop-2-yn-1-yl)-1H-benzo[d]imidazol-2-yl]pyridine 0.144-hydrate Geometric parameters (Å, º)

**Table:**

O1A—H1A	0.8700	C9—H9	0.9500
O1A—H1B	0.8697	C9—C10	1.382 (2)
O1B—H1C	0.8704	C10—H10	0.9500
O1B—H1D	0.8701	C10—C11	1.401 (2)
N1—C1	1.3441 (18)	C11—H11	0.9500
N1—C5	1.3456 (18)	C11—C12	1.384 (2)
N2—C6	1.3804 (16)	C12—H12	0.9500
N2—C7	1.3850 (18)	C13—H13A	0.9900
N2—C13	1.4650 (17)	C13—H13B	0.9900
N3—C6	1.3257 (18)	C13—C14	1.4654 (19)
N3—C8	1.3841 (19)	C14—C15	1.182 (2)
N4—C16	1.3821 (17)	C15—H15	0.9500
N4—C17	1.3805 (18)	C17—C18	1.403 (2)
N4—C23	1.4616 (16)	C17—C22	1.389 (2)
N5—C16	1.3231 (18)	C18—C19	1.396 (2)
N5—C18	1.379 (2)	C19—H19	0.9500
C1—C2	1.4022 (19)	C19—C20	1.372 (3)
C1—C16	1.473 (2)	C20—H20	0.9500
C2—H2	0.9500	C20—C21	1.401 (2)
C2—C3	1.370 (2)	C21—H21	0.9500
C3—H3	0.9500	C21—C22	1.383 (2)
C3—C4	1.380 (2)	C22—H22	0.9500
C4—H4	0.9500	C23—H23A	0.9900
C4—C5	1.3975 (19)	C23—H23B	0.9900
C5—C6	1.472 (2)	C23—C24	1.4722 (18)
C7—C8	1.3995 (19)	C24—C25	1.185 (2)
C7—C12	1.393 (2)	C25—H25	0.9500
C8—C9	1.394 (2)		
			
H9···O1A^i^	2.16	C12···C14	3.376 (2)
H4···O1A^ii^	2.62	C16···C17^vi^	3.3783 (19)
O1A···H19	2.36	C1···H23B	2.89
O1B···H1D	0.87	H1A···C4^iv^	2.85
H9···O1B^i^	2.15	C5···H13B	2.87
H13A···O1B^iii^	2.37	C13···H23B	2.86
O1B···H1C	0.87	C14···H23B	2.78
N1···N4	2.9842 (16)	C18···H1B	2.56
N1···C13	3.0587 (18)	C18···H1D	2.60
N1···N2	3.0092 (16)	C19···H1B	2.38
N1···C23	3.012 (2)	C19···H1D	2.64
N1···H23B	2.34	C22···H23A	2.87
N1···H13B	2.36	C23···H13B	2.85
H1A···N3^iv^	2.14	C24···H13B	2.74
N3···H25^v^	2.46	H1A···H19	2.37
H1C···N3^iv^	2.21	H4···H1A^ii^	1.96
N3···H4	2.44	H1B···H19	1.83
N5···H2	2.45	H4···H1C^ii^	2.37
N5···H1B	2.37	H1C···H1D	1.38
N5···H1D	2.24	H1D···H19	2.21
C2···C22^vi^	3.381 (2)	H13B···H23B	2.19
C5···C21^vi^	3.392 (2)	H19···H1A	2.37
C5···C20^vi^	3.386 (2)		
			
H1A—O1A—H1B	104.5	C12—C11—C10	121.92 (15)
H1C—O1B—H1D	104.5	C12—C11—H11	119.0
C1—N1—C5	117.30 (11)	C7—C12—H12	121.9
C6—N2—C7	106.65 (11)	C11—C12—C7	116.18 (14)
C6—N2—C13	130.75 (12)	C11—C12—H12	121.9
C7—N2—C13	122.50 (11)	N2—C13—H13A	109.3
C6—N3—C8	105.92 (11)	N2—C13—H13B	109.3
C16—N4—C23	130.94 (12)	N2—C13—C14	111.68 (11)
C17—N4—C16	106.62 (11)	H13A—C13—H13B	107.9
C17—N4—C23	122.40 (11)	C14—C13—H13A	109.3
C16—N5—C18	105.49 (12)	C14—C13—H13B	109.3
N1—C1—C2	122.79 (14)	C15—C14—C13	177.08 (16)
N1—C1—C16	120.28 (12)	C14—C15—H15	180.0
C2—C1—C16	116.88 (13)	N4—C16—C1	127.05 (12)
C1—C2—H2	120.6	N5—C16—N4	112.28 (13)
C3—C2—C1	118.89 (15)	N5—C16—C1	120.61 (12)
C3—C2—H2	120.6	N4—C17—C18	105.48 (13)
C2—C3—H3	120.3	N4—C17—C22	131.78 (13)
C2—C3—C4	119.33 (14)	C22—C17—C18	122.73 (14)
C4—C3—H3	120.3	N5—C18—C17	110.13 (13)
C3—C4—H4	120.7	N5—C18—C19	130.10 (14)
C3—C4—C5	118.62 (15)	C19—C18—C17	119.75 (15)
C5—C4—H4	120.7	C18—C19—H19	121.1
N1—C5—C4	123.03 (14)	C20—C19—C18	117.73 (15)
N1—C5—C6	120.51 (11)	C20—C19—H19	121.1
C4—C5—C6	116.44 (13)	C19—C20—H20	119.0
N2—C6—C5	127.57 (12)	C19—C20—C21	121.95 (15)
N3—C6—N2	111.95 (13)	C21—C20—H20	119.0
N3—C6—C5	120.47 (12)	C20—C21—H21	119.3
N2—C7—C8	105.87 (12)	C22—C21—C20	121.39 (16)
N2—C7—C12	131.57 (13)	C22—C21—H21	119.3
C12—C7—C8	122.56 (14)	C17—C22—H22	121.8
N3—C8—C7	109.61 (13)	C21—C22—C17	116.43 (15)
N3—C8—C9	130.06 (14)	C21—C22—H22	121.8
C9—C8—C7	120.32 (14)	N4—C23—H23A	109.3
C8—C9—H9	121.2	N4—C23—H23B	109.3
C10—C9—C8	117.58 (15)	N4—C23—C24	111.62 (11)
C10—C9—H9	121.2	H23A—C23—H23B	108.0
C9—C10—H10	119.3	C24—C23—H23A	109.3
C9—C10—C11	121.43 (15)	C24—C23—H23B	109.3
C11—C10—H10	119.3	C25—C24—C23	179.18 (16)
C10—C11—H11	119.0	C24—C25—H25	180.0
			
N1—C1—C2—C3	−2.3 (2)	C8—N3—C6—N2	0.42 (15)
N1—C1—C16—N4	3.0 (2)	C8—N3—C6—C5	−178.67 (11)
N1—C1—C16—N5	−179.95 (12)	C8—C7—C12—C11	−1.4 (2)
N1—C5—C6—N2	13.2 (2)	C8—C9—C10—C11	−0.9 (2)
N1—C5—C6—N3	−167.82 (12)	C9—C10—C11—C12	0.6 (2)
N2—C7—C8—N3	0.31 (15)	C10—C11—C12—C7	0.5 (2)
N2—C7—C8—C9	−178.53 (12)	C12—C7—C8—N3	179.96 (12)
N2—C7—C12—C11	178.18 (13)	C12—C7—C8—C9	1.1 (2)
N3—C8—C9—C10	−178.54 (14)	C13—N2—C6—N3	−176.46 (12)
N4—C17—C18—N5	0.41 (15)	C13—N2—C6—C5	2.6 (2)
N4—C17—C18—C19	−178.00 (12)	C13—N2—C7—C8	176.56 (11)
N4—C17—C22—C21	178.90 (13)	C13—N2—C7—C12	−3.1 (2)
N5—C18—C19—C20	−179.42 (14)	C16—N4—C17—C18	−0.70 (14)
C1—N1—C5—C4	−0.05 (19)	C16—N4—C17—C22	−179.75 (14)
C1—N1—C5—C6	178.26 (11)	C16—N4—C23—C24	−102.55 (15)
C1—C2—C3—C4	0.7 (2)	C16—N5—C18—C17	0.06 (15)
C2—C1—C16—N4	−174.45 (13)	C16—N5—C18—C19	178.26 (14)
C2—C1—C16—N5	2.62 (19)	C16—C1—C2—C3	175.04 (14)
C2—C3—C4—C5	1.1 (2)	C17—N4—C16—N5	0.79 (15)
C3—C4—C5—N1	−1.5 (2)	C17—N4—C16—C1	178.07 (12)
C3—C4—C5—C6	−179.86 (13)	C17—N4—C23—C24	80.13 (15)
C4—C5—C6—N2	−168.33 (13)	C17—C18—C19—C20	−1.4 (2)
C4—C5—C6—N3	10.61 (19)	C18—N5—C16—N4	−0.53 (15)
C5—N1—C1—C2	1.97 (19)	C18—N5—C16—C1	−178.00 (11)
C5—N1—C1—C16	−175.30 (11)	C18—C17—C22—C21	0.0 (2)
C6—N2—C7—C8	−0.05 (14)	C18—C19—C20—C21	0.5 (2)
C6—N2—C7—C12	−179.67 (14)	C19—C20—C21—C22	0.6 (2)
C6—N2—C13—C14	−113.23 (15)	C20—C21—C22—C17	−0.9 (2)
C6—N3—C8—C7	−0.45 (15)	C22—C17—C18—N5	179.57 (12)
C6—N3—C8—C9	178.24 (14)	C22—C17—C18—C19	1.2 (2)
C7—N2—C6—N3	−0.23 (15)	C23—N4—C16—N5	−176.85 (12)
C7—N2—C6—C5	178.78 (12)	C23—N4—C16—C1	0.4 (2)
C7—N2—C13—C14	71.06 (15)	C23—N4—C17—C18	177.19 (11)
C7—C8—C9—C10	0.0 (2)	C23—N4—C17—C22	−1.9 (2)

Symmetry codes: (i) −*x*+1/2, *y*−1/2, −*z*+3/2; (ii) *x*−1/2, −*y*+3/2, *z*+1/2; (iii) −*x*+3/2, *y*−1/2, −*z*+3/2; (iv) *x*+1/2, −*y*+3/2, *z*−1/2; (v) *x*−1, *y*, *z*; (vi) −*x*+1, −*y*+1, −*z*+1.

### 2,6-Bis[1-(prop-2-yn-1-yl)-1H-benzo[d]imidazol-2-yl]pyridine 0.144-hydrate Hydrogen-bond geometry (Å, º)

**Table:**

*D*—H···*A*	*D*—H	H···*A*	*D*···*A*	*D*—H···*A*
O1*A*—H1*A*···N3^iv^	0.87	2.14	2.923 (16)	149
O1*A*—H1*B*···N5	0.87	2.37	3.178 (17)	154
O1*B*—H1*C*···N3^iv^	0.87	2.21	2.96 (3)	144
O1*B*—H1*D*···N5	0.87	2.24	3.07 (4)	161
C9—H9···O1*A*^i^	0.95	2.16	2.891 (15)	133
C9—H9···O1*B*^i^	0.95	2.15	2.93 (3)	139
C19—H19···O1*A*	0.95	2.36	3.086 (18)	133

Symmetry codes: (i) −*x*+1/2, *y*−1/2, −*z*+3/2; (iv) *x*+1/2, −*y*+3/2, *z*−1/2.
